# Henry Charles Lea

**Published:** 1909-12

**Authors:** 


Henry Charles Lea, widely known as an author, banker, scientist and publisher, died recently at his home in Philadelphia, 84 years old. He had been ill from pneumonia for less than a week. Mr. Lea was probably best known from his writings on the history of the Middle Ages and on church subjects. At the time of his death he was preparing a work on the history of the Spanish Inquisition. He was engaged in the publishing business from 1843 to 1880. He retired in order to devote more time to literature.

